# Effect of strip shelterwood-cuts on the crown morphology plasticity of natural regenerated *Pinus tabuliformis* saplings in northeastern China

**DOI:** 10.7717/peerj.9826

**Published:** 2020-08-26

**Authors:** Huilin Gao, Jian Feng, You Yin, Wanjin Hu, Yang Qu, Mingguo Liu

**Affiliations:** 1 College of Forestry, Shenyang Agricultural University, Shenyang, Liaoning Province, China; 2 Chinese Forest Ecosystem Research Network, Research Station of Liaohe-River Plain Forest Ecosystem, Tieling, Liaoning Province, China; 3 Liaoning Academy of Forestry Sciences, Shenyang, Liaoning Province, China; 4 Liaoning Ecological Experimental Forest Farm, Chaoyang, Liaoning Province, China

**Keywords:** Natural regeneration sapling, Branch analysis, Outermost crown profile, Annual growth of branch

## Abstract

The study analysed the effect of shelterwood-cut strips on the outermost crown profiles and crown characteristics of naturally regenerated *Pinus tabuliformis* saplings in northeastern China. A total of 49 regenerated saplings from shelterwood-cut strips and 30 from uncut strips were collected. Nonlinear quantile regression was used to develop the outermost crown profile model for the saplings from the shelterwood-cut and uncut strips. The quantile value suitable for describing the outermost crown profiles of the two types of strips was selected using nonparametric boundary regression. The difference in crown morphologies between the shelterwood-cut strips and uncut strips was compared. The results showed that with the same diameter at breast height, the crown radii of the uncut strip saplings were larger than those of the shelterwood-cut strip saplings within the range of 0.2–1.0 for the relative depth into the crown. The largest crown radius of the saplings from the uncut strips was larger than that of the saplings from the shelterwood-cut strips. The inflection points of the shelterwood-cut strip sapling crowns were larger than those of the uncut strip sapling crowns. The crown volume of the small uncut strip saplings was larger than that of the shelterwood-cut strip saplings, and the difference in crown volume decreased with increasing sapling size. The saplings in the early stage of the uncut strips showed a greater growth rate than those of the shelterwood-cut strips, but their growth rate slowed over the long term according to branch-length annual growth. The present study provides a reference for forest management strategy decision making in promoting natural regeneration.

## Introduction

Shelterwood-cut strips have generally been an effective method to establish adequate regeneration after harvesting given their natural conservation benefits and advantages in promoting sustainable forest management (Magalí et al., 2019). With higher coniferous regeneration and high stock, shelterwood-cut strips usually offer a more effective alternative to other methods in terms of silvicultural and technical and organisational feasibility (Pominville & Rucl, 1995; Hlásny et al., 2017). The growth of saplings in shelterwood-cut strips is important in evaluating the effectiveness of shelterwood-cut strips (Williams, Messier & Kneeshaw, 1999; Mori, Fukasawa & Takeda, 2008; Valkonen et al., 2011). Therefore, it is necessary to pay attention to the growth of saplings from shelterwood-cut strips.

Crowns play an essential role in the productivity efficiency of individual trees since crowns serve as the location of photosynthesis, respiration and transpiration (Colin & Houllier, 1992; Crecente-Campo et al., 2009; Gao, Bi & Li, 2017). The crown morphology plasticity of a sapling influences both light capture and the use of growth resources and may reflect important implications related to the ability of a sapling to conduct efficient photosynthesis in the understory of a light-limiting environment and to compete with neighbours (Williams, Messier & Kneeshaw, 1999; Nikinmaa et al., 2003; Navarro-Cerrillo et al., 2018). For conifer saplings, crown morphology plasticity has been described by a series of characteristics in terms of the number of branches per whorl, the ratio of leader height to lateral branch growth, and the crown depth or crown ratio (Williams, Messier & Kneeshaw, 1999; Sattler & Lemay, 2001; Nemec, Parish & Goudie, 2002; Saud et al., 2016). The crown morphology of conifer saplings varies from a conical crown form to a more flat-topped form in the forest understory under different light conditions (Pearcy & Yang, 1998). Shade-tolerant saplings generally invest more photosynthetic products in the lateral expansion of a crown to increase light interception, and light-demanding saplings invest more in height growth to avoid being shaded by neighbours (Mori & Takeda, 2004). The trade-off in the forest understory between lateral expansion and height growth determines survival and future competitive advantages for saplings.

The crown profile is a suitable characteristic for reflecting the horizontal and vertical crown dimensions of an individual tree and is also useful for reflecting the crown morphology plasticity of saplings (Crecente-Campo et al., 2009; Baldwin & Peterson, 1997). However, unlike the adult trees that reach the upper canopy, the crown profiles of shade-grown saplings are always irregular and are difficult to describe due to the substantially varied microenvironments in the forest understory (Doruska & Mays, 1998; Dong et al., 2015). Thus, in comparison to adult trees, shade saplings should balance photosynthate allocation between horizontal expansion for more light capture and height growth to avoid being shaded by the crown of neighbouring trees (Mori & Takeda, 2004). An accurate description of sapling crown profiles is informative for evaluating the growth status of saplings.

The parametric regression approach has been widely used in crown profile modelling (Baldwin & Peterson, 1997; Crecente-Campo et al., 2009; Gao, Bi & Li, 2017; Gao, Dong & Li, 2017; Sun, Gao & Li, 2017). Generally, parametric regression uses an empirically developed equation, and easily measured tree variables (e.g. diameter at breast height (DBH), height to DBH ratio (HD), and crown radius (CR)) are used as independent variables (Crecente-Campo et al., 2009; Gao, Bi & Li, 2017; Gao, Dong & Li, 2017). Quantile regression provides an estimation of the conditional quantile function at any probability level with few constraints on data distribution (Koenker & Bassett, 1978; Koenker, 2015; Gao, Bi & Li, 2017). However, the crown profile described by quantile regression is not the outermost contour of the exterior edges of the crown (Gao, Bi & Li, 2017). Nonparametric regression analysis, a data-driven technique with much more adapted data and fewer restrictions compared to those of the parametric approach, is powerful for describing the boundary curve of a data cloud. Thus, nonparametric boundary regression is favourable for selecting the specific quantile to model the outermost crown profiles of saplings for the quantile regression approach. Crown profiles of adult trees have already been well studied. However, to the best of our knowledge, the outermost crown profiles of saplings from shelterwood-cut strips have not been well studied, and the effect of shelterwood-cut strips on crown morphology plasticityis still unclear. Quantile regression and nonparametric boundary regression have not been used in outermost crown-profile modelling for saplings.

*Pinus tabuliformis* performs well in terms of conserving water and soil and protecting the environment (Wang & Liu, 2011). Planted *Pinus tabuliformis* trees cover an area of approximately 0.14 million hectares in Liaoning Province, northeastern China. The area of mature *Pinus tabuliformis* plantations accounts for approximately 65% of the total area of this tree species. Studying the effect of shelterwood-cut strips on the crown morphology plasticity of naturally regenerating *Pinus tabuliformis* saplings will provide a reference for forest management decision making. The specific purposes of this study are (1) to develop an outermost crown profile model for saplings from shelterwood-cut strips (sun-grown saplings) and uncut strips (shade-grown saplings) using nonlinear quantile regression, with nonparametric boundary regression being used to select the quantile value to model the outermost crown profiles of saplings, and (2) to compare the differences in the crown morphology plasticities of saplings including largest crown radius, inflection points, crown volume of upper crown and annual growth of branch length between the shelterwood-cut and uncut strips.

## Materials and Methods

### Study area

The research was conducted at Liaoning ecological and experimental farms located in the semiarid areas of the western part of Liaoning Province, northeastern China (120°15′–121°18′E, 41°23′–42°17′N). The area has a continental monsoon climate in the northern temperate zone. The mean air temperature of this area is 8.3 °C, with a maximum temperature of 40.7 °C and minimal temperature of −26.4 °C with in a year. The mean annual precipitation ranges from 450 mm to 550 mm, with approximately 70% distributed within July and August. The elevation ranges from 200 m to 1074 m, and the frost-free period is usually 145–150 days. Brown forest soil is the main soil type in this area.

**Figure 1:**
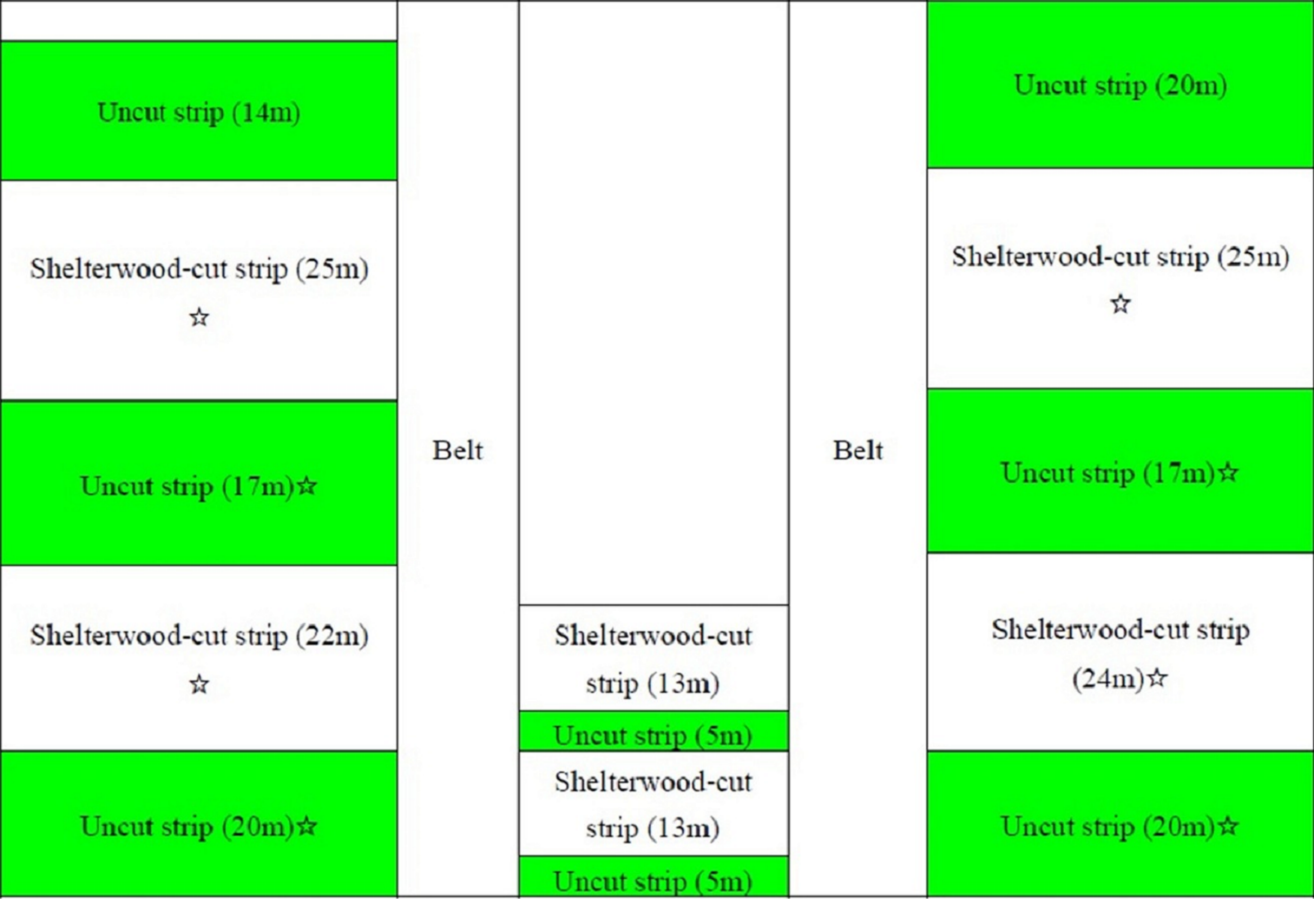
Scheme of the shelterwood-cut strips in the *Pinus tabuliformis* stand in the western part of Liaoning Province. Strips with green and white colours indicate uncut and shelterwood-cut strips, respectively; the ☆ indicates the strip where sample saplings were obtained.

Our study area is located in the mature *Pinus tabuliformis* plantation that is 45 years old that has a density of 1,035 trees per hectare in the Linghe experimental region of the Liaoning ecological and experimental farm. In the spring of 2014, the shelterwood-cut strip experiment was conducted. Part of the framework of the research is shown in Fig. 1. The widths of the shelterwood-cut strips varied from 13 to 25 m, and the widths of the uncut stand strips were 5–20 m. Timber and slash were removed immediately after the strip-cut process. The surface soil and the regenerated saplings were not destroyed during the strip-cut work. To reduce the effect of strip width on our results, all the measurements were taken from shelterwood-cut strips with widths of 22 m and 25 m and from uncut strips with widths of 17 m and 22 m. The orientation of the shelterwood-cut strips were in the south–north direction. The average tree height of the mature pine was 15.9 m. All the saplings in this area naturally regenerated within the stands.

### Data collection

At the end of the growing season in November 2017, a total of 50 saplings from the shelterwood-cut strip and 30 saplings from the uncut strip were selected. All the saplings were selected from the 2-m wide belts from the centre of the shelterwood-cut and uncut strips to ensure homogeneous conditions regarding light availability for the saplings from shelterwood-cut and uncut strips. For all saplings, the total tree height (HT, 0.1 m) was greater than 1.3 m, and the diameter at breast height (0.1 cm) was less than 5.0 cm. HT and DBH were measured before felling for all the sampled saplings (Table 1).

**Table 1:** Descriptive statistics of the tree and branch attributes for the 49 saplings from the shelterwood-cut strips and 30 from the uncut strips. *N* denotes the sample size; Std denotes the standard deviation.

Strip types	Characteristics	Variables	Mean	Std	Min	Max
Shelterwood-cut strip	Saplings (*N* = 49)	DBH (cm)	2.46	0.99	0.71	4.70
		HT (m)	2.40	0.59	1.38	3.83
		CL (m)	1.85	0.45	1.01	2.74
	All branches (*N* = 1,398)	BL (cm)	45	28	1	146
		BC (cm)	41	25	1	136
		VA (°)	62	17	5	160
		BD (mm)	8.40	3.73	1.28	26.91
Uncut strip	Saplings (*N* = 30)	DBH (cm)	1.36	0.54	0.57	3.17
		HT (m)	2.18	0.49	1.30	3.27
		CL (m)	1.61	0.36	1.01	2.34
	All branches (*N* = 753)	BL (cm)	39	24	1	105
		BC (cm)	36	22	1	95
		VA (°)	73	14	8	115
		BD (mm)	5.50	2.17	1.68	12.82

**Note:**

DBH, diameter breast height; HT, total tree height; CL, crown length; BL, branch length; BC, branch chord length; VA, branch angle; BD, branch diameter.

**Figure 2:**
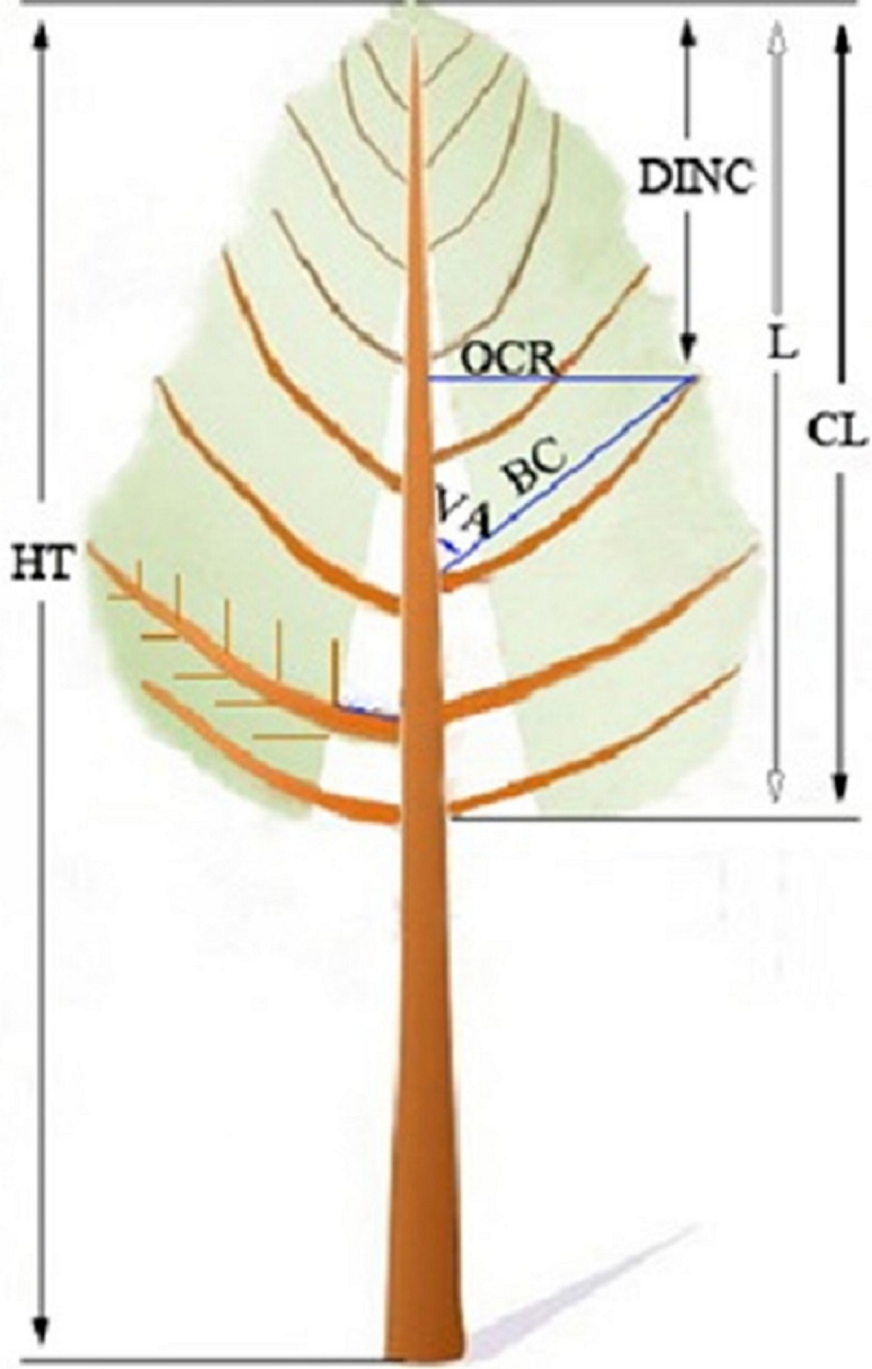
An illustrative sketch of the branch and sapling characteristics of the naturally regenerated *Pinus tabuliformis* saplings from shelterwood-cut strips and uncut strips. HT is total height, CL is crown length, L is the length from sapling tip to the branch base, DINC is the depth into the crown radius of interest, RDINC = DINC/CL, OCR is outer crown radius, BC is branch chord length, and VA is branch angle (Gao, Dong & Li, 2017).

For the sampled saplings, the whorl containing at least one branch that was continuous to the former whorl was defined as the crown base, and the length from the sapling tip to the crown base was the crown length (CL). A straight line from the ground level to breast height was marked on the trunk to indicate the north side, which facilitated the determination of the azimuth of the branches, and the line was extended to the sapling tip after the saplings were carefully felled. After felling, the HTs of the saplings were remeasured. To facilitate the measurement, the saplings were divided into 1-m sections, while any section less than 1 m was considered a sapling tip. Each section was placed upright on the ground. For each branch from each whorl from every sapling, the vertical distance along the trunk from the branch base to the sapling tip (L) was measured first. Then, the branch diameter (BD), branch length (BL), branch chord length (BC), branch angle (VA) and azimuth, which denotes the angle deviated from the north side in the clockwise direction, were measured (Fig. 2). To detect the effect of shelterwood-cut strips on the annual growth of branch length, one 5-year-old sample branch with from the fifth whorl was selected from each sapling. The annual growth of the branch length for all sampled branches was measured.

To detect possible recording or measurement errors in the field work, a visual inspection of the crown profiles was carefully implemented for each sapling. One specific sapling from the shelterwood-cut strip with a DBH of 0.73 cm and a tree height of 1.4 m showed a strange profile and was considered biologically unreasonable. Thus, this sapling was removed from the data set, leaving 49 saplings from the shelterwood-cut strips with a total of 507 whorls and 1,398 branches for the analysis and modelling. For the 30 saplings from the uncut strips, a total of 363 whorls and 753 branches were harvested. The absolute depth into the crown radius of interest (DINC) was calculated as the difference between the distances from the sapling tip to the branch base and the projection of the branch onto the trunk. The relative depth into the crown radius of interest (RDINC) was calculated as RDINC = DINC/CL. The outermost crown radius (OCR) was calculated as the branch chord length multiplied by the sine value of the branch angle. The characteristics of the saplings and branches from the shelterwood-cut strips and uncut strips are shown in Table 1.

### Parametric regression approach for the crown profile model

For the parametric regression approach, the models that perform well with other species when targeting crown profile, largest branch radius distribution and taper description of adult trees were selected as the candidate models. Due to the irregularity of the crown profiles for the saplings, the models used in the description of the crown profiles of the adult trees were reconstructed in the present study (Roeh & Maguire, 1997; Gao et al., 2019).

The power-exponential equation performs well in modelling the *Pinus sylvestris* var. Mongolica plantation and was used in the present study (Sun, Gao & Li, 2017). The basic form of the power-exponential equation is shown in Eq. (1).

(1) $${\rm OCR} = {{a}_{\rm 1}} \cdot {\rm RDIN}{{\rm C}^{{{a}_{\rm 2}}}}{{\rm e}^{{{a}_{\rm 3}} \cdot {\rm RDINC}}}$$

where OCR is the outermost crown radius within the crown, RDINC is the relative depth into the crown of interest, and *a*_1_–*a*_3_ are the model parameters to be estimated.

The Kozak equation is a continuous variable-exponential equation with a changing exponent from the ground to the top of a tree that describes the different shapes within the entire bole (Kozak, 1988). Due to its flexibility, it has been modified to be applied to the trend in branch diameter within acrown and the crown profile description (Maguire, Johnston & Cahill, 1999; Weiskittel et al., 2010; Gao, Bi & Li, 2017). The basic model form is shown in Eq. (2).

(2) $${\rm OCR} = {{c}_{\rm 1}} \cdot {{X}^{{{c}_{\rm 2}}}} = {{c}_{\rm 1}} \cdot {\left[ {\displaystyle{{{\rm 1 - (1 - RDINC}{{\rm )}^{0.5}}} \over {{\rm 1 - }{{p}^{0.5}}}}} \right]^{{c}_{\rm 2}}}$$

where the base *X* is a function of RDINC, the point of inflection *p* and the two other parameters *c*_1_ and *c*_2_ are variables to be estimated. The OCR is zero at the tip of the tree when RDINC equals zero and reaches the maximum, that is the largest crown radius at the point of inflection when RDINC equals *p*.

Both basic models (Eqs. (1) and (2)) were further modified or tested in terms of their performance in crown profile modelling for the saplings. Ordinary least square estimation (OLS) was used to estimate the model parameters on the R platform (R Core Team, 2018). The dataset including the branch with the largest radius from each whorl was used to fit these candidate models. The goodness-of-fit statistics of adjusted *R*^2^ (*R*_adj_^2^) and root of mean square error (RMSE) (Eqs. (3) and (4)) were used to compare the models. The model with the largest *R*_adj_^2^ and the smallest RMSE was selected as the best model to describe the outermost crown profiles of the *Pinus tabuliformis* saplings.

(3) $${{R}_{{\rm adj}}}^{{\hskip-9pt}\rm 2} {\hskip4pt}= {\rm 1} - \left( {{\rm 1} - {{R}^{\rm 2}}} \right)\left( {\displaystyle{{{n} - {\rm 1}} \over {{n} - {p}}}} \right) = {\rm 1} - {\rm SSE}\left( {\displaystyle{{{n} - {\rm 1}} \over {{\rm SST}}}} \right)$$

(4) $${\rm RMSE} = \sqrt {\displaystyle{{\sum\limits_{{i} = {\rm 1}}^{M} {\sum\limits_{{j} = {\rm 1}}^{{{n}_{i}}} {{{{\rm (OC}{{\rm R}_{{ij}}}-{{ {\rm O}}}{\hat{\rm C}}{{\rm R}_{{ij}}}{\rm )}}^{\rm 2}}} } } \over {{n - 1}}}}$$

where ${{R}^{\rm 2}} = {\rm 1} - \sum\limits_{{i} = {\rm 1}}^{M} {\sum\limits_{{j} = {\rm 1}}^{{{n}_{i}}} {{{{\rm (OC}{{\rm R}_{{ij}}}-{{\rm O}}{\hat {\rm C}{\rm R}_{{ij}}}{\rm )}}^{\rm 2}}{\rm /}\sum\limits_{{i} = {\rm 1}}^{M} {\sum\limits_{{j} = {\rm 1}}^{{{n}_{i}}} {{{{\rm (OC}{{\rm R}_{{ij}}}-{{\rm O}}{{\bar {\rm C}{\rm R}}_{{ij}}}{\rm )}}^{\rm 2}}} } } }$, OCR*_ij_* is the outer crown radius of the *j*th branch from the *i*th sapling, ${{\rm O}}{\hat {\rm C}{\rm R}_{{ij}}}$ is the predicted outermost crown radius of the *j*th branch from the *i*th sapling, *M* is the number of saplings, and *n_i_* is the number of branches from the *i*th sapling.

Nonlinear quantile regression with an interior point algorithm proposed by Koenker & Park (1996) was used to model the outermost crown profiles. The general form of the nonlinear quantile regression model is shown in Eq. (5), and the nonlinear quantile regression estimator was defined as in Eq. (6) (Koenker, 2015).

(5) $${{Q}_{{{Y}_{i}}}}{\rm (}\left. {\rm \tau } \right|{{x}_{i}}{\rm )} = {g(}{{x}_{i}}{\rm ,}{{\rm \beta }_{\rm 0}}{\rm (}{\rm \tau}))$$

(6) $${{\hat {\rm \beta}}_{n}}({\rm \tau} = {\rm argmin}{_{{b} \in {B}}}\sum {{{\rm \rho }_{\rm \tau }}} {\rm (}{{y}_{i}} - {g(}{{x}_{i}}{,b))}$$

The estimation for the parameters of the specific quantile was implemented by the quantreg package of R software (Koenker, 2018). In the present study, the outermost crown profile model using a nonlinear quantile regression approach with the same deterministic nonlinear function is shown in Eq. (7).

(7) $${\rm OC}{{\rm R}_{{ij}}} = {f(X,{\rm{\theta}} )} + {{\rm \varepsilon }_{{ij}}}$$

where OCR*_ij_* is the outer crown radius of the *j*th branch from the *i*th sapling, *f*(·) is the nonlinear equation form, *X* represents the variables, θ represents the parameters, and ε*_ij_* is the error term.

### Outermost crown profile description for the saplings

A nonlinear quantile regression model was used to describe the outermost crown profiles of the *Pinus tabuliformis* saplings from the shelterwood-cut and uncut strips. Nonparametric boundary regression with a polynomial frontier estimator was used to determine the specific quantile for the nonlinear quantile regression to model the outermost crown profiles. For the nonparametric boundary regression approach, the data pairs (RDINC*_i_*, OCR*_i_*) where *i* indicates the number of branches per tree were used to characterise the branch tips. A single polynomial equation with a known degree parameter *p* was used to address the full observations as follows (Abdelaati, Thibault & Hohsuk, 2017):

(8) $${\rm OCR} = {\varphi _{\rm \theta }}{\rm (RDINC)} = {{\rm \theta }_{\rm 0}} + {{\rm \theta }_{\rm 1}} \cdot {\rm RDINC} + \cdots + {{\rm \theta }_{p}} \cdot {\rm RDINC}^{p}\; {\rm RDINC} \in {[a,b]}$$

where *a* and *b* are the lower and upper endpoints, respectively, of the design points RDINC_1_,…, RDINC_*n*_, where *n* indicates the number of divisions for the interval of [*a*, *b*]. In the present study, *a* equalled 0, and *b* equalled 1 according to the biological characteristics of the crown. The optimal polynomial degree was obtained by minimising AIC and BIC. The nonparametric boundary estimator and the optimal polynomial degree were estimated by the new R software package ‘npbr’ (Abdelaati, Thibault & Hohsuk, 2017; Koenker, 2018).

A set of quantile values ranging from 0.5 to 0.99 with an even interval of 0.01 was specified for the nonlinear quantile regression model. Nonparametric boundary regression was used to describe the outermost crown profiles for all the saplings from the shelterwood-cut strips and uncut strips. RDINC was divided into 10,000 intervals by an even interval of 0.0001. The crown radius of all the specified RDINCs was calculated for each sapling by a series of nonlinear quantile regression models and nonparametric boundary regression. The absolute distance between the two curves modelled by nonlinear quantile regression and nonparametric boundary regression for each sapling was calculated (Gao, Bi & Li, 2017). The specific nonlinear quantile regression model with the smallest distance to the nonparametric boundary was selected to describe the outermost crown profile for each sapling (Gao, Bi & Li, 2017). Finally, the quantiles used to describe the outermost crown profiles of the saplings for the shelterwood-cut strips and uncut strips were calculated by averaging all the outermost quantiles for the two types of strips.

### Effect of shelterwood-cut strips on the crown morphology plasticity of saplings

The outermost crown profile model developed by the nonlinear quantile regression approach was used to depict the crown morphology plasticity of the saplings from shelterwood-cut and uncut strips. The inflection points were calculated for each sapling. The difference in the inflection points of the saplings from shelterwood-cut strips and uncut strips was compared. The crown volume for the upper crown was calculated by a rotational integral using the predicted outermost crown profile curves. The crown morphology plasticity, including the largest crown radius, inflection points, and annual growth of branch length of the two types of saplings, was also compared.

## Results

### Basic model selection for the outermost crown profile

All parameters of the power-exponential equation and Kozak equation (Eqs. (1) and (2)) were reparameterized to the sapling variables. *R*_adj_^2^ and RMSE were used to compare different models, and the best model was selected for each basic equation, as shown in Eqs. (9) and (10).

For the power-exponential equation, *a*_1_, *a*_2_, and *a*_3_ were reparameterized by introducing easily measured sapling variables. The final model form is shown in Eq. (9).

(9) $${\rm OCR} = {{a}_{\rm 1}} \cdot {\rm DB}{{\rm H}^{{{a}_{\rm 2}}}}{\rm RDIN}{{\rm C}^{{\rm (}{{a}_{\rm 3}} + {{a}_{\rm 4}} \cdot {\rm CR)}}}{\rm exp(}{{a}_{\rm 5}} \cdot {\rm RDINC)}$$

where OCR is the outermost crown radius within the crown, DBH is the diameter at breast height, CR is the crown ratio, *a*_1_–*a*_5_ are the parameters to be estimated, and the other variables are defined as those above.

For the Kozak equation, we further modified the basic equation to fit the crown profiles of the saplings, and it is shown as follows (Eq. (10)):

(10) $${\rm OCR} = {c_1} \cdot {\rm DBH}^{c_2} \cdot \left({\frac{1-(1-{\rm RDINC})^{0.5}}{1-({c_3} \cdot {\rm CR}^{c_{4}})^{0.5}}}\right)^{({c_5} \cdot (1-{\rm RDINC}))}$$

where *c*_1_–*c*_5_ are the parameters to be estimated, and the other variables were defined as those above.

Parameter estimates and goodness-of-fit statistics for Eqs. (9) and (10) are shown in Table 2. The *R*_adj_^2^ for the power-exponential equation and the modified Kozak equation with only DBH and CR as independent variables was 0.62. The power-exponential equation was much more flexible in describing the outermost crown radius and was thus selected as the best model to describe the outermost crown profiles for the *Pinus tabuliformis* saplings.

**Table 2:** Goodness-of-fit statistics for the crown profile model for the natural regeneration of *Pinus tabuliformis* saplings based on the power-exponential equation, segmented polynomial equation, and modified Kozak equation.

Models	Variables included	Number of parameters	*R*_adj_^2^	RMSE
Power-exponential equation	DBH, CR	5	0.62	0.1435
Modified Kozak equation	DBH, CR	5	0.62	0.1432

### Outermost crown profile model development using nonlinear quantile regression

The power-exponential equation was used as the basic equation to model the outermost crown profiles for the *Pinus tabuliformis* saplings. The nonlinear quantile regression models were developed for a set of quantile values from 0.5 to 0.99 with an even interval of 0.01 for the saplings from the shelterwood-cut strip and uncut strip. All sapling branches from the shelterwood-cut strip and uncut strip were used to estimate the model parameters. The estimates of the parameters for the 0.75, 0.85, 0.95, and 0.99 quantiles of the shelterwood-cut and uncut strips are shown in Table 3.

**Table 3:** Parameter estimates for the ordinary regression and nonlinear quantile regression crown profile model for the natural regeneration of *Pinus tabuliformis* saplings from shelterwood-cut and uncut strips.

Strip types	Quantiles	Types	*a*_1_	*a*_2_	*a*_3_	*a*_4_	*a*_5_
Shelterwood-cut strip	Ordinary least square	Estimates	0.3848	0.6002	1.3131	−0.4378	−1.6558
		Std	0.0706	0.0339	0.1529	0.1675	0.1680
	*q* = 0.75	Estimates	0.3543	0.5363	1.2589	−0.5247	−1.1415
		Std	0.0623	0.0342	0.1050	0.1146	0.1459
	*q* = 0.85	Estimates	0.3881	0.4892	1.2204	−0.5747	−0.9509
		Std	0.0484	0.0247	0.1021	0.1109	0.1195
	*q* = 0.95	Estimates	0.5648	0.3607	1.0896	−0.5414	−0.7556
		Std	0.1194	0.0508	0.1046	0.1388	0.1468
	*q* = 0.99	Estimates	0.6379	0.2500	0.8087	−0.4039	−0.3635
		Std	0.1532	0.0382	0.1199	0.1313	0.2319
	*q* = 0.88	Estimates	0.4030	0.4751	1.1811	−0.5538	−0.9125
		Std	0.0574	0.0241	0.1160	0.1306	0.1426
Uncut strip	Ordinary least square	Estimates	1.1287	0.5288	1.4591	−0.0522	−2.3823
		Std	0.3423	0.0458	0.1673	0.0750	0.3096
	*q* = 0.75	Estimates	0.6884	0.5346	1.1902	−0.0289	−1.6889
		Std	0.1478	0.0442	0.0916	0.0592	0.1804
	*q* = 0.85	Estimates	0.8041	0.4941	1.1184	0.0141	−1.6638
		Std	0.1992	0.0462	0.1466	0.1139	0.2086
	*q* = 0.95	Estimates	1.2189	0.2915	1.0042	−0.0639	−1.3446
		Std	0.6316	0.0723	0.2289	0.2042	0.4596
	*q* = 0.99	Estimates	0.9989	0.3235	0.8906	−0.0947	−0.9920
		Std	0.6651	0.0852	0.2951	0.2252	0.4998
	*q* = 0.92	Estimates	1.2834	0.3950	1.2543	−0.1097	−1.8092
		Std	0.3302	0.0550	0.1456	0.1245	0.2696

**Note:**

*q* is the abbreviation for quantile.

The results indicated that the crown curves based on the nonlinear quantile regression with the same quantile value for the saplings from shelterwood-cut and uncut strips were different in terms of parameter estimates. The distances between the predicted crown profiles from the nonlinear quantile regression with crown quantiles from 0.50 to 0.99 with aneven interval of 0.01 and the fitted outermost crown profiles by nonparametric boundary regression were calculated for all saplings. For the shelterwood-cut strip saplings, the nonlinear quantile regression crown profile model with the quantile of 0.88 had the smallest distance with the nonparametric boundary regression approach. For the uncut strip saplings, the smallest distance was in the 0.92 quantile. The estimates for the two nonlinear quantile regression models for the outermost crown profilesare shown in Table 3 and were used to compare crown morphology plasticity.

### Comparison of crown morphology for saplings between shelterwood-cut and uncut strips

**Figure 3:**
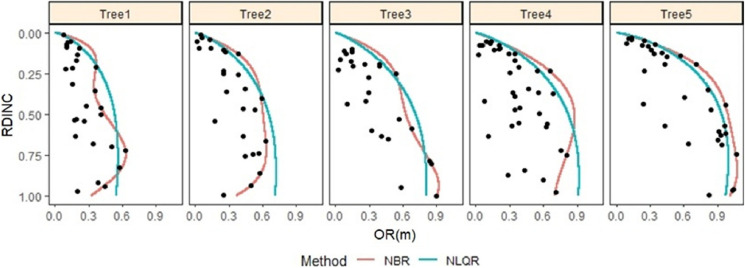
Outermost crown profile description for five randomly selected naturally regenerated *Pinus tabuliformis* saplings from the shelterwood-cut strips predicted using NBR and NLQR and compared against the vertical trend of the observed crown radius within. OR is crown radius, RDINC is relative depth into the crown of interest, NBR is nonparametric boundary regression, NLQR is nonlinear quantile regression, and the black dot is the observed crown radius. For tree 1, DBH = 1.29 cm and CR = 0.89; for tree 2, DBH = 2.27 cm and CR = 0.73; for tree 3, DBH = 2.94 cm and CR = 0.63; for tree 4, DBH = 3.82 cm and CR = 0.68; and for tree 5, DBH = 4.38 cm and CR = 0.85.

**Figure 4:**
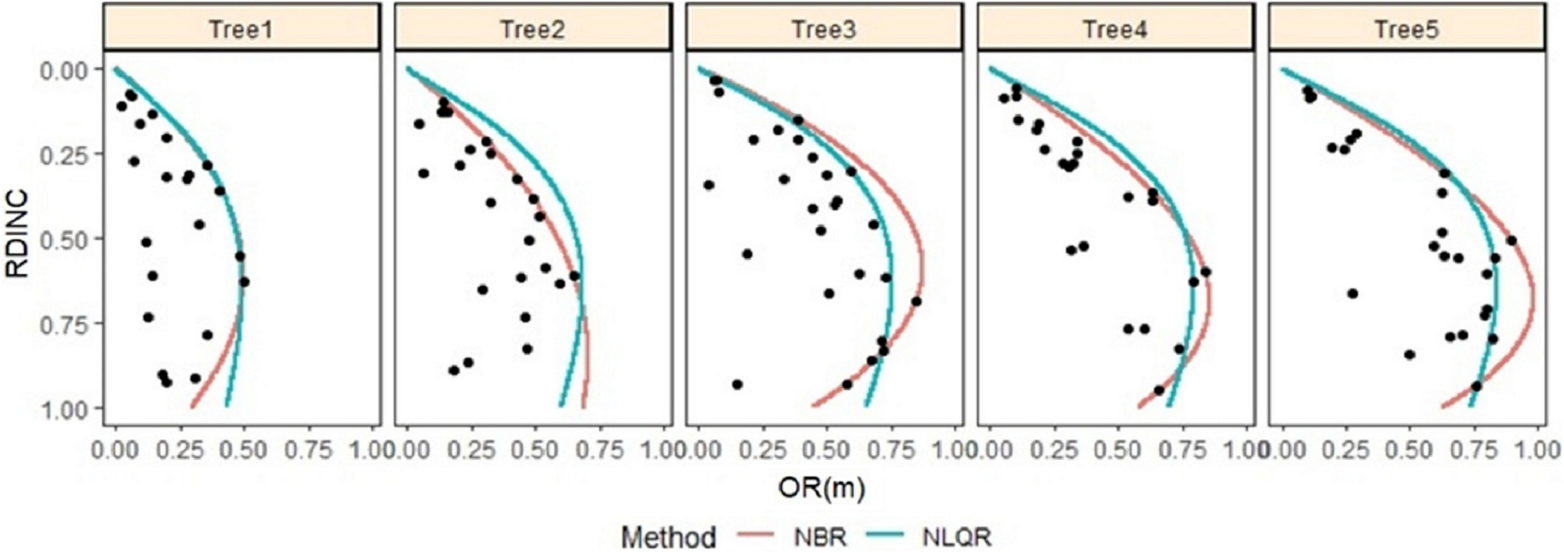
Outermost crown profile description for five randomly selected naturally regenerated *Pinus tabuliformis* saplings from the uncut strips predicted using NBR and NLQR and compared against the vertical trend of the observed crown radius within the crown. For tree 1, DBH = 0.62 cm and CR = 0.78; for tree 2, DBH = 1.42 cm and CR = 0.82; for tree 3, DBH = 1.77 cm and CR = 0.63; for tree 4, DBH = 2.08 cm and CR = 0.77; and for tree 5, DBH = 2.39 cm and CR = 0.77.

With the nonparametric boundary regression, the nonlinear quantile regression was powerful and suitable in describing and predicting the outermost crown profile for the individual saplings from both the shelterwood-cut (Fig. 3) and uncut strips (Fig. 4). Thus, nonlinear quantile regression was used in determining the crown morphology plasticity for the two types of saplings.

**Figure 5:**
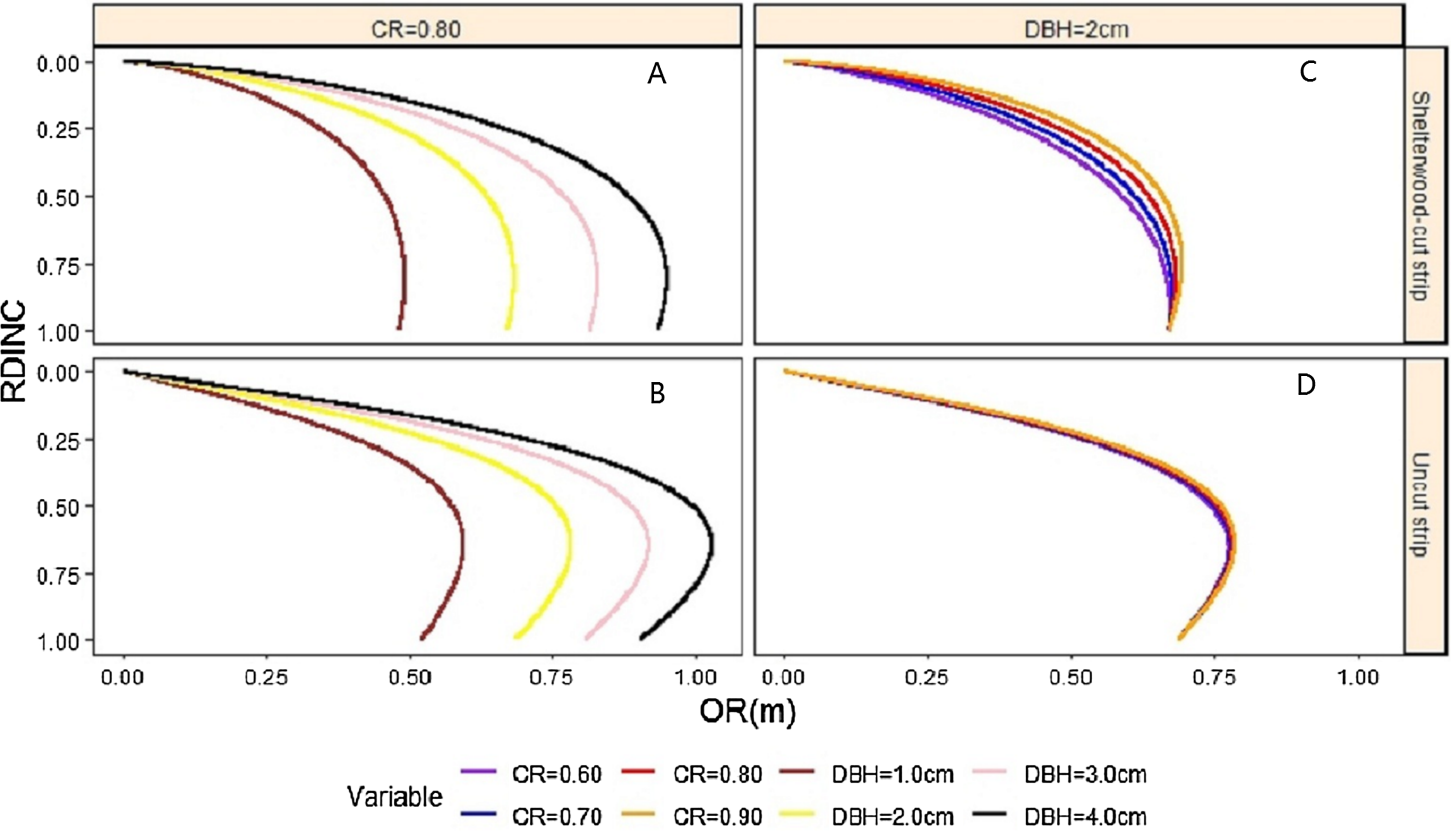
Effects of crown ratio (CR) (A and B) and diameter at breast height (DBH) (C and D) on the crown profiles of *Pinus tabuliformis* saplings from the shelterwood-cut strips and uncut strips.

The relationship between the characteristics of the saplings and crown regularity was compared between the shelterwood-cut strip and uncut strip saplings. The CR was fixed at 0.80, approximately the mean CR value of our sampled saplings, and the relationship between the crown radius and DBH for the two strip types was analysed (Figs. 5A and 5B). Similarly, the relationship between the CR and crown radius for the saplings from the two strip types is shown in Figs. 5C and 5D, when DBH was fixed at the approximate mean value of 2.0 cm. Under the condition of fixing CR, the crown radius increased with increasing DBH for all saplings from the shelterwood-cut and uncut strips. The crown radius values of the shelterwood-cut saplings werelarger than those of the uncut saplings within the range of 0–0.2 for the RDINC. The crown radius values in which the RDINC were larger than 0.20 for the saplings from the shelterwood-cut strips were smaller than the crown radius values from the uncut strip, and the difference decreased with increasing DBH. The crown radius values increased with increasing CR when the DBH was fixed, while the CR had a much more significant effect on the shelterwood-cut strip sapling crowns than on the uncut strip sapling crowns (Figs. 5C and 5D). With increasing CR, the largest crown radius for both shelterwood-cut strip and uncut strip saplings only slightly increased.

The crown characteristics of the saplings from shelterwood-cut strips and uncut strips were analysed. For the largest crown radius, saplings from the uncut strips were larger than those of the saplings from the shelterwood-cut strips when the saplings from the two strips were the same size (Figs. 5A and 5B). The inflection points of the shelterwood-cut strip sapling crowns were larger than those of the uncut strip sapling crowns. The mean value of the inflection points was 0.81 for the shelterwood-cut strip saplings and 0.65 for the uncut strip saplings. The crown volume of the small uncut strip saplings was larger than that of the shelterwood-cut strip saplings of the same size, and the difference decreased with increasing sapling size.

**Figure 6:**
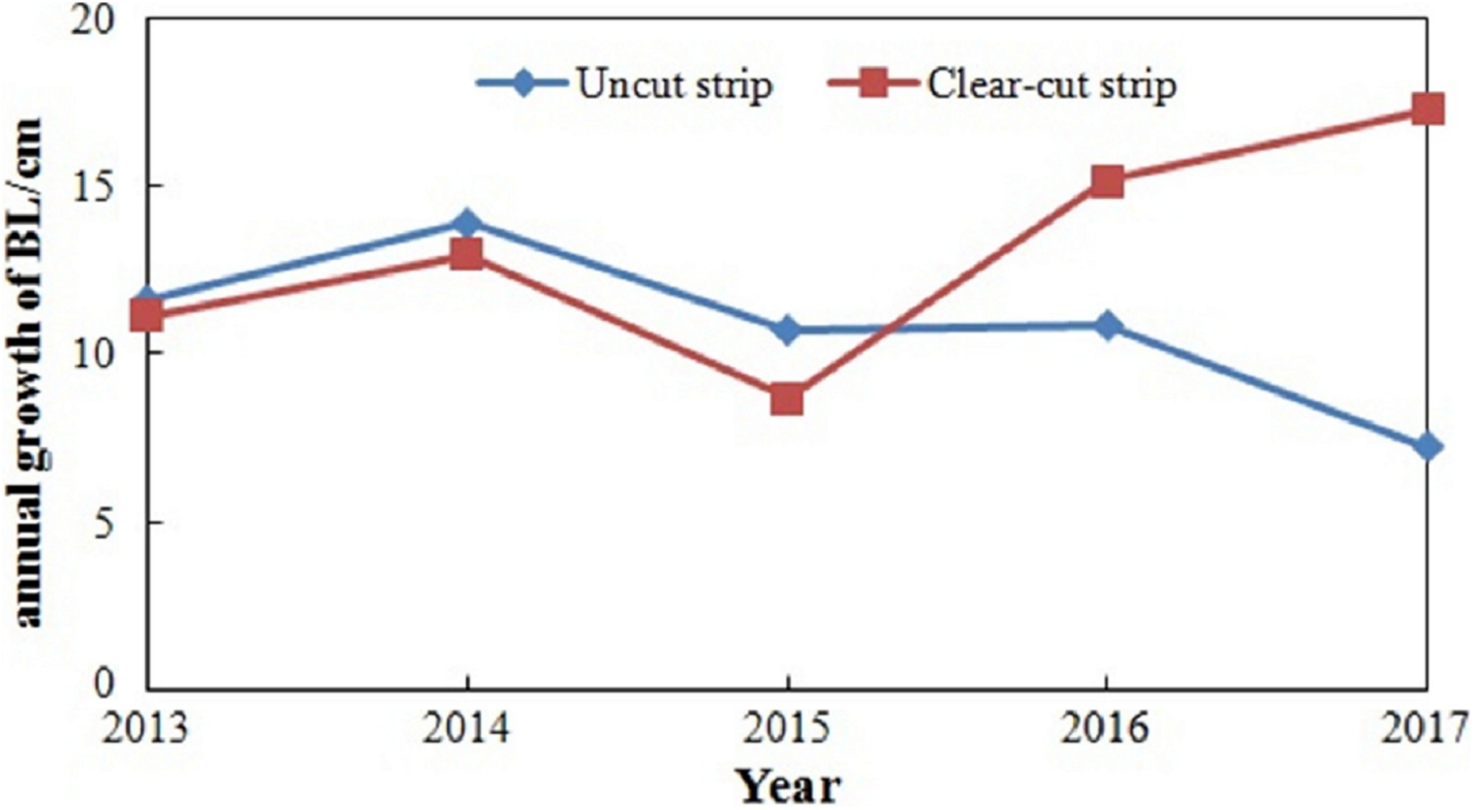
Annual growth of the branch length of the saplings from the shelterwood-cut strips and uncut strips due to the implementation of the shelterwood-cut strip experiment from 2014 to 2017.

The annual growth of branch lengths for the saplings from the shelterwood-cut strips and uncut strips were analysed and compared, as shown in Fig. 6. In 2013, before the implementation of the shelterwood-cut strip experiment, the mean annual growth of branch length for shelterwood-cut strip saplings was 11.2 cm, and that of the uncut strip saplings was 11.5 cm. Under similar growing conditions, there was almost no difference in the annual growth of branch length between the two types of saplings. In 2014, the mean annual growth of branch length was 12.9 cm and 14.0 cm for the shelterwood-cut strip and uncut strip saplings, respectively, and the annual growth of branch length for the uncut strip saplings was significantly larger than that for the shelterwood-cut strip saplings. The differences in annual growth between the two types of saplings continued to increase until 2015. However, there was no significant difference in the annual growth of branch length before 2015 (*p* > 0.05). After 2015, the growth rate of the shelterwood-cut strip saplings increased and was significantly larger than that of the uncut strip saplings (*p* < 0.05).

## Discussion

Shelterwood-cut strips are an effective method for developing adequate natural regeneration (Pothier, 2000; Hidding, Tremblay & Côté, 2012; Russell, Westfall & Woodall, 2017; Kenzo, Yoneda & Ninomiya, 2018). Generally, approximately 12–15% of the total variation in the rate of regeneration contributes to the differences in stands and shelterwood-cut strips (Pliūra, Kundrotas & Suchockas, 2000). Crown morphology plasticity is a desirable sapling characteristic, and analysing crown morphology plasticity of saplings from shelterwood-cut strips and uncut strips is essential in evaluating the effect of shelterwood-cut strips on natural regeneration (Biging & Dobbertin, 1992; Mclaughlin, Wullschleger & Nosal, 2003; Crecente-Campo et al., 2009; Daouia, Laurent & Noh, 2017). To ensure homogeneous growing conditions regarding light availability, all saplings were randomly selected, but the belts maintained at a 2-m width in the centre of each shelterwood-cut strip or uncut strip. Pliūra, Kundrotas & Suchockas (2000) revealed that only a weak negative correlation exists between the regeneration of spruce and the width of strips. Therefore, the result of our study was not influenced by the difference in strip width between the shelterwood-cut and uncut strips.

Generally, in comparison to sun-grown saplings, shade-grown saplings invest more in lateral growth to capture more light because light is the limiting factor. In contrast to shade-grown saplings, sun-grown saplings invest more in height growth than in lateral expansion because competition is the main limiting factor (Huber, May & Hülsbergen, 2016). For shade- and sun-grown saplings, the trade-off between lateral crown expansion and height growth should be considered because as more is invested in lateral crown expansion, less light interception occurs in the understory, and as more is invested in height growth, less shade occurs from competitors (Mori & Takeda, 2004). In the present study, the largest crown radius for the uncut strip saplings was larger than that of the shelterwood-cut strip saplings for saplings of the same size. This result clearly indicated that saplings of the same size invested more in lateral growth so that the crown increased its surface area to intercept more light (Fig. 5). Therefore, light interception is an essential factor in determining crown morphology.

The inflection points for the shelterwood-cut strip saplings were larger than those of the uncut strip saplings. The combination of the largest crown radius and inflection point determined the difference in the crown morphology of the saplings from the shelterwood-cut strips and uncut strips. This was the reason why the crown morphology of the uncut strip saplings was flatter, and the crown morphology of the shelterwood-cut strip saplings was similar to a parabola. The growth rate of the saplings was an essential factor affecting the crown morphology (Claveau et al., 2002). Therefore, the annual growth for the branch length within the period of conduction for the shelterwood-cut strip experiment was analysed to explain the difference in the crown morphology plasticity for the two types of saplings. The annual growth of the branch length for the shelterwood-cut strip saplings was lower than that of the uncut strip saplings, and the difference was larger 2 years after the implementation of the shelterwood-cut strips. Thus, the saplings in the early stage of the uncut strip showed a greater growth rate than that of the shelterwood-cut strip saplings but slowed their growth rate over the long term. Obviously, these findings would affect silvicultural decisions. Within one or 2 years after the implementation of shelterwood-cut strips, protection measures should be taken for shelterwood-cut strip saplings, such as stopping destruction from wildlife.

The effect of DBH and CR on the outermost crown profiles of the saplings from the two types of strips was different. We found that DBH had a significant effect on the outermost crown profiles of both the shelterwood-cut and uncut strip saplings. However, the CR significantly affected only the shelterwood-cut strip saplings but not the uncut strip saplings. This result indicated that non-functional branches would not affect the growth of saplings from the uncut strip. This process is important to the management of natural regeneration.

Some studies have shown that light-demanding tree species are able to grow in a shaded understory, although at a slow rate (Oberbauer et al., 1993; Niinemets & Lukjanova, 2003; Noémie et al., 2011). We also reached the same conclusion, and the growth rate was even greater at the early stage than at the late stage for the shaded saplings. Therefore, the time at which the shelterwood-cut strips were conducted was essential to the growth of saplings. A number of studies have also shown that sapling growth is influenced by sapling size independent of any effect on light availability (Niinemets & Lukjanova, 2003; Claveau, Messier & Comeau, 2005). With increasing sapling size, the proportion of non-photosynthetic tissue increases faster than that of photosynthetic tissue (Oberbauer et al., 1993). In comparison to smaller saplings, larger saplings might require substantially more light to maintain respiration and growth (Oberbauer et al., 1993; Pearcy & Yang, 1998). Thus, the conclusions of this study will provide useful information on the link between growth rate and crown size for *Pinus tabuliformis* saplings to develop effective management strategies to improve the growth of *Pinus tabuliformis* saplings in the understory. However, the sampled saplings in the present study came from only one location and the accuracy and generality of the results may be limited to some extent. In addition, a full light control was also meaningful to further analyse the effect of strip shelterwood-cuts on the crown morphology plasticity of the saplings and it was also not considered in the present study. In the next step, we will further add the quantitative light measurement and full light control in our experiment and enlarge the study area to make our results more generalised.

## Conclusions

Crown morphology plasticity of the naturally regenerating saplings of *Pinus tabuliformis* from shelterwood-cut strips and uncut strips was analysed and compared in northeastern China. Nonlinear quantile regression modelled the outermost crown profiles of the saplings from both the shelterwood-cut and uncut strips well. DBH affected the outermost crown profiles of the saplings from the shelterwood-cut and uncut strips. In contrast, CR affected the outermost crown profiles of the saplings from the shelterwood-cut strips but not from the uncut strips. The saplings from shelterwood-cut strips generally had the smaller largest crown radius and larger inflection point than those from the uncut strips. The crown volume of the small uncut strip saplings was larger than that of the shelterwood-cut strip saplings of the same size, and the volume decreased with increasing sapling size. The saplings from the uncut strips tended to maintain a flat crown shape to intercept more light for the small saplings. With increasing sapling size, the sapling from the uncut strips tended to grow more slowly due to severely limited light interception.

## Supplemental Information

10.7717/peerj.9826/supp-1 Supplemental Information 1 Sapling characteristics and branch length measurement. A total of 49 and 30 saplings from clear-cut and uncut strip were provided. The sapling sample branch was selected from each whorl of each saplings were measured for the annual branch length.

## Additional Information and Declarations

### Data Availability

The following information was supplied regarding data availability: The raw data are available as a Supplemental File.
